# A New Functional Role for Mechanistic/Mammalian Target of Rapamycin Complex 1 (mTORC1) in the Circadian Regulation of L-Type Voltage-Gated Calcium Channels in Avian Cone Photoreceptors

**DOI:** 10.1371/journal.pone.0073315

**Published:** 2013-08-19

**Authors:** Cathy Chia-Yu Huang, Michael Lee Ko, Gladys Yi-Ping Ko

**Affiliations:** Department of Veterinary Integrative Biosciences, College of Veterinary Medicine and Biomedical Sciences, Texas A&M University, College Station, Texas, United States of America; Yale School of Medicine, United States of America

## Abstract

In the retina, the L-type voltage-gated calcium channels (L-VGCCs) are responsible for neurotransmitter release from photoreceptors and are under circadian regulation. Both the current densities and protein expression of L-VGCCs are significantly higher at night than during the day. However, the underlying mechanisms of circadian regulation of L-VGCCs in the retina are not completely understood. In this study, we demonstrated that the mechanistic/mammalian target of rapamycin complex (mTORC) signaling pathway participated in the circadian phase-dependent modulation of L-VGCCs. The activities of the mTOR cascade, from mTORC1 to its downstream targets, displayed circadian oscillations throughout the course of a day. Disruption of mTORC1 signaling dampened the L-VGCC current densities, as well as the protein expression of L-VGCCs at night. The decrease of L-VGCCs at night by mTORC1 inhibition was in part due to a reduction of L-VGCCα1 subunit translocation from the cytosol to the plasma membrane. Finally, we showed that mTORC1 was downstream of the phosphatidylionositol 3 kinase-protein kinase B (PI3K-AKT) signaling pathway. Taken together, mTORC1 signaling played a role in the circadian regulation of L-VGCCs, in part through regulation of ion channel trafficking and translocation, which brings to light a new functional role for mTORC1: the modulation of ion channel activities.

## Introduction

The mechanistic/mammalian target of rapamycin (mTOR) signaling pathway governs diverse cellular physiological functions including cell growth, cell survival, energy balance, and metabolism in response to environmental signals such as nutrients and stress [1–6]. mTOR, a conserved serine (ser) / threonine (thr) protein kinase, is composed of two distinct complexes, namely mTOR complex 1 (mTORC1) and mTORC2. mTORC1 regulates cell growth by increasing protein synthesis through phosphorylation of downstream targets, p70 ribosomal S6 kinase (p70S6K) and eukaryotic translation initiation factor 4E-binding protein 1(4EBP1) [7–11], while mTORC2 regulates cell survival and cytoskeletal organization [12–15]. In the retina, mTOR signaling is important for cell survival and axon regeneration. Stimulation of mTOR signaling by insulin prolongs the survival of retinal neurons [16,17], and depletion of the negative regulators of mTOR promotes axon regeneration in retinal ganglion cells after optic nerve injury [18,19]. Under hyperglycemic conditions, the suppression of mTOR activity in diabetic retinas causes apoptosis [20]. Therefore, the mTOR signaling pathway is essential for maintaining retinal metabolic homeostasis and health.

While mTOR is essential in metabolism and cell survival, it is also involved in the circadian regulation of both vertebrates and invertebrates [21–23]. The circadian clocks regulate metabolism, physiological processes, and behaviors across the course of a day, and these internal time-keeping mechanisms allow organisms to anticipate and adapt to daily external environmental changes such as cycling ambient illumination and temperature fluctuations [24,25]. The canonical core mechanism underlying the circadian oscillations is composed of a specific set of “clock genes” and their protein products, which form self-regulated transcriptional-translational feedback loops with a period close to 24 hours [24,25]. However, other post-translational mechanisms such as phosphorylation, methylation, and ubiquitination, as well as various cellular signaling pathways are also involved in the circadian mechanism or the circadian regulation of downstream targets [26]. mTOR signaling is involved with the core circadian oscillator components and affects the rhythmicity. Disruption of mTOR signaling alters the light-induced expression of the *Period* gene, a core oscillator component [22], as well as light-induced phase shifting in animal activity rhythm [22], while activation of mTOR signaling impacts the nuclear accumulation of the clock protein TIMELESS and lengthens the circadian period in *Drosophila* [21]. Hence, mTOR signaling may participate in the core circadian mechanism.

In the vertebrate retina, many physiological aspects are under circadian control, since the visual system has to adapt to large changes in ambient illumination throughout the day [27,28]. In particular, the circadian oscillators in retinal photoreceptors regulate daily changes in retinomotor movement [29,30], outer segment shedding and renewal [31], gene and protein expression [32–35]; morphological changes at synaptic ribbons [36], as well as ion channel activities [37,38]. We previously showed a circadian regulation of L-type voltage-gated calcium channels (L-VGCCs) in cone photoreceptors [38]. The L-VGCCs are essential for neurotransmitter release from photoreceptors and other retinal neurons [39]. We further demonstrated that both Ras-mitogen-activated protein kinase (MAPK) and Ras-phosphatidylionositol 3 kinase-protein kinase B (PI3K-AKT) signaling pathways are part of the circadian output pathway mediating L-VGCC trafficking and insertion in a circadian phase-dependent manner [38,40]. Since mTOR is involved in the circadian mechanism, we investigated whether it also participates as part of the circadian output pathway to regulate L-VGCCs in cone photoreceptors. We combined biochemical, morphological, and electrophysiological analyses to examine the potential circadian phase-dependent modulation of L-VGCCs by mTOR and its potential interaction with other signaling pathways.

## Experimental Procedure

### Cell cultures and circadian entrainment

Fertilized eggs (*Gallus gallus*) were obtained from the Poultry Science Department, Texas A&M University (College Station, TX, USA). Chicken retinas were dissociated at embryonic day 12 (E12) and cultured for 6 days as described previously [38,40]. Cultures were prepared in the presence of 20 ng/ml ciliary neurotrophic factor (CNTF; R&D Systems, Minneapolis, MN, USA), which yields cultures highly enriched with cone photoreceptors [41–43] and 10% heat-inactivated horse serum. Cell culture incubators (maintained at 39°C and 5% CO_2_) were equipped with lights and timers, which allowed for the entrainment of retinal circadian oscillators to 12h: 12h light-dark (LD) cycles *in vitro*. Zeitgeber time zero (ZT 0) was designated as the time when the lights turned on and ZT 12 was the time when the lights went off. For *in ovo* entrainment, intact eggs were exposed to LD 12h: 12h at E10-E11 for 7 days. Retina cells were then dissociated, cultured, kept in constant darkness (DD), and used for biochemical and molecular biological assays on the second day of DD. In some experiments, after *in ovo* LD entrainment for 6 days, eggs were kept in DD. On the second day of DD, retinas were collected at different circadian time (CT) points throughout a day for biochemical assays [38,40]. The reason for using chick embryos from E12+6 for *in vitro* entrainment or E18 for *in ovo* entrainment is that more than 90% of the retina photoreceptors express functionally mature VGCC currents by E18 [44].

### Immunoblot analysis

Samples were collected and prepared as described previously [45]. Briefly, intact retinas were homogenized in Tris lysis buffer including (in mM): 50 Tris,1 EGTA, 150 NaCl, 1% Triton X-100, 1% β-mercaptoethanol, 50 NaF, 1 Na _3_VO_4_; pH 7.5. Samples were separated on 10% sodium dodecyl sulfate–polyacrylamide gels by electrophoresis and transferred to nitrocellulose membranes. The primary antibodies used in this study were: anti-di-phospho-ERK (pERK; Sigma, St. Louis, MO, USA), anti- ERK (total ERK, used for loading control; Santa Cruz Biochemicals, Santa Cruz, CA, USA), anti-VGCCα1D (Alomone, Jerusalem, Israel), anti-phospho-mTORC1 (ser2448; Cell Signaling Technology, Danvers, MA, USA), anti-phospho-mTORC1 (ser2481; Cell Signaling Technology), anti-mTORC (total mTORC, Cell Signaling Technology), anti-phospho-S6 (ser240/244; Cell Signaling Technology), anti-S6 (Cell Signaling Technology), anti-phospho-p70S6K (thr389; Cell Signaling Technology), and anti-p70S6K (Cell Signaling Technology). Blots were visualized using appropriate secondary antibodies conjugated to horseradish peroxidase (Cell Signaling Technology) and an enhanced chemiluminescence (ECL) detection system (Pierce, Rockford, IL, USA). Relative protein expressions for all proteins involved in this study are reported as a ratio to total ERK since total ERK remains constant throughout the day. Band intensities were quantified by densitometry using Scion Image (NIH, Bethesda, MD, USA). All measurements were repeated at least 3 times.

### Electrophysiology

Whole cell patch-clamp configuration of L-VGCC current recordings were carried out using mechanically ruptured patches. For retinal photoreceptors, the external solution was (in mM): 110 NaCl, 10 BaCl_2_, 0.4 MgCl_2_, 5.3 KCl, 20 TEA-Cl, 10 HEPES, and 5.6 glucose, pH 7.35 with NaOH. The pipette solution was (in mM): 135 Cs acetate, 10 CsCl, 1 NaCl, 2 MgCl_2_, 0.1 CaCl_2_, 1.1 EGTA, and 10 HEPES, pH 7.3 adjusted with CsOH. Recordings were made only from cells with elongated cell bodies with one or more prominent oil droplets (hallmark of avian cone photoreceptors) [33,37,44]. Currents were recorded at room temperature (RT, 23°C) using an Axopatch 200B (Axon Instruments/Molecular Devices, Union City, CA, USA) or A-M 2400 amplifier (A-M Systems Inc., Carlsborg, WA, USA). Signals were low-pass filtered at 2 kHz and digitized at 5 kHz with Digidata 1440A interface and pCLAMP 10.0 software (Molecular Devices). Electrode capacitance was compensated after gigaohm (GΩ) seals were formed. Cells were held at -65mV, and ramp voltage commands from -80 to +60 mV in 500 ms were used to evoke Ba^2+^ currents. Current–voltage (I–V) relations were also elicited from a holding potential of -65 mV in 200 ms steps (5 s between steps) to test potentials over a range of -80 to +60 mV in 10 mV increments. The maximal currents were obtained when the steps depolarized to 0 ~ +10 mV. The membrane capacitance, series resistance, and input resistance of the recorded photoreceptors were measured by applying a 5 mV (100 ms) depolarizing voltage step from a holding potential of -65 mV. Cells with an input resistance smaller than 1 GΩ were discarded. The membrane capacitance reading was used as the value for whole cell capacitance. The current densities (pA/pF) were obtained by dividing current amplitudes by membrane capacitances. Rapamycin and PP242 were obtained from A.G. Scientific (San Diego, CA, USA) and Chemdea (Ridgewood, NJ, USA), respectively. Both rapamycin and PP242 were dissolved in DMSO (the final concentration of DMSO vehicle was 0.1%).

### Calcineurin activity assay

Retina samples were lysed in a phosphatase lysis buffer including (in mM): 50 Tris, pH 7.5, 1 EGTA, 150 NaCl, 1% Triton X-100, and 1% β-mercaptoethanol, and calcineurin activities were assayed using a commercially available ser/thr phosphatase assay kit (Promega, Madison, WI, USA). This kit can distinguish between tyrosine (tyr) and ser/thr phosphatases by using a synthetic polypeptide, RRA(pT) VA, that is compatible with ser/thr phosphatases but is structurally incompatible for tyr phosphatases. To differentiate between PP2A, 2B, and 2C, the reaction buffer is made to favor one over the others since this class of enzyme has a diverse range of optimum conditions. For calcineurin (PP2B), the reaction buffer contained 250 mM imidazole (pH 7.2), 1 mM EGTA, 50 mM MgCl_2_, 5 mM NiCl_2_, 250 µg/ml calmodulin, and 0.1% β-mercaptoethanol as described in the manufacturer’s protocol. Free cytoplasmic phosphate was first removed from the samples then dephosphorylation of the kit’s calcineurin substrate proceeded for 30 min at RT. This system determines the amount of free phosphate generated in a reaction by measuring the absorbance (600 nm) of a molybdate/ malachite green/ phosphate complex.

### Immunocytochemistry

Dissociated retinas were cultured on coverslips and entrained under LD cycle for four days. Cell were then fixed at CT4 or CT16 with Zamboni fixative for 30 min at RT, washed in phosphate buffer (0.1M PB, pH7.4), and permeabilized in 1% Triton-X PB for 10min. Samples were blocked in 10% goat serum in 0.1% Triton-X/PB for 2 hr at RT, then incubated with VGCCα1D primary antibody (1:100) overnight. The cells were washed with 0.1% Triton-X/PB and incubated with fluorescent conjugated secondary antibody (Alexa 488nm goat anti-rabbit; Molecular Probes, Carlsbad, CA, USA) for 2 hr in the dark. Coverslips were then re-washed and mounted with ProLong® Gold antifade reagents with 4',6-diamidino-2-phenylindole (DAPI; Invitrogen, Eugene, OR, USA) on a glass slide and stored at 4°C for later observation on a Zeiss microscope (Thornwood, NY, USA) with epi-fluorescence to determine the localization of VGCCα1D and the nucleus (with DAPI). Green or blue fluorescent images were taken under identical settings including exposure time and magnification. The fluorescence intensity was measured using Adobe Photoshop 12 software (Adobe Systems, San Jose, CA, USA) as described previously [38]. The fluorescence intensity analyses were carried out blindly. The experiment was repeated at least four times.

### Statistical analysis

All data are presented as mean ± SEM (standard error of mean). One-way analysis of variance (ANOVA) followed by Tukey’s post hoc test for unbalanced n was used for statistical analyses. Throughout, * *p*<0.05 was regarded as significant. Any defined rhythmic expression had to exhibit at least a 1.5 fold change in rhythmic amplitude [46].

## Results

### mTORC1 signaling is under circadian control

**Figure 1:**
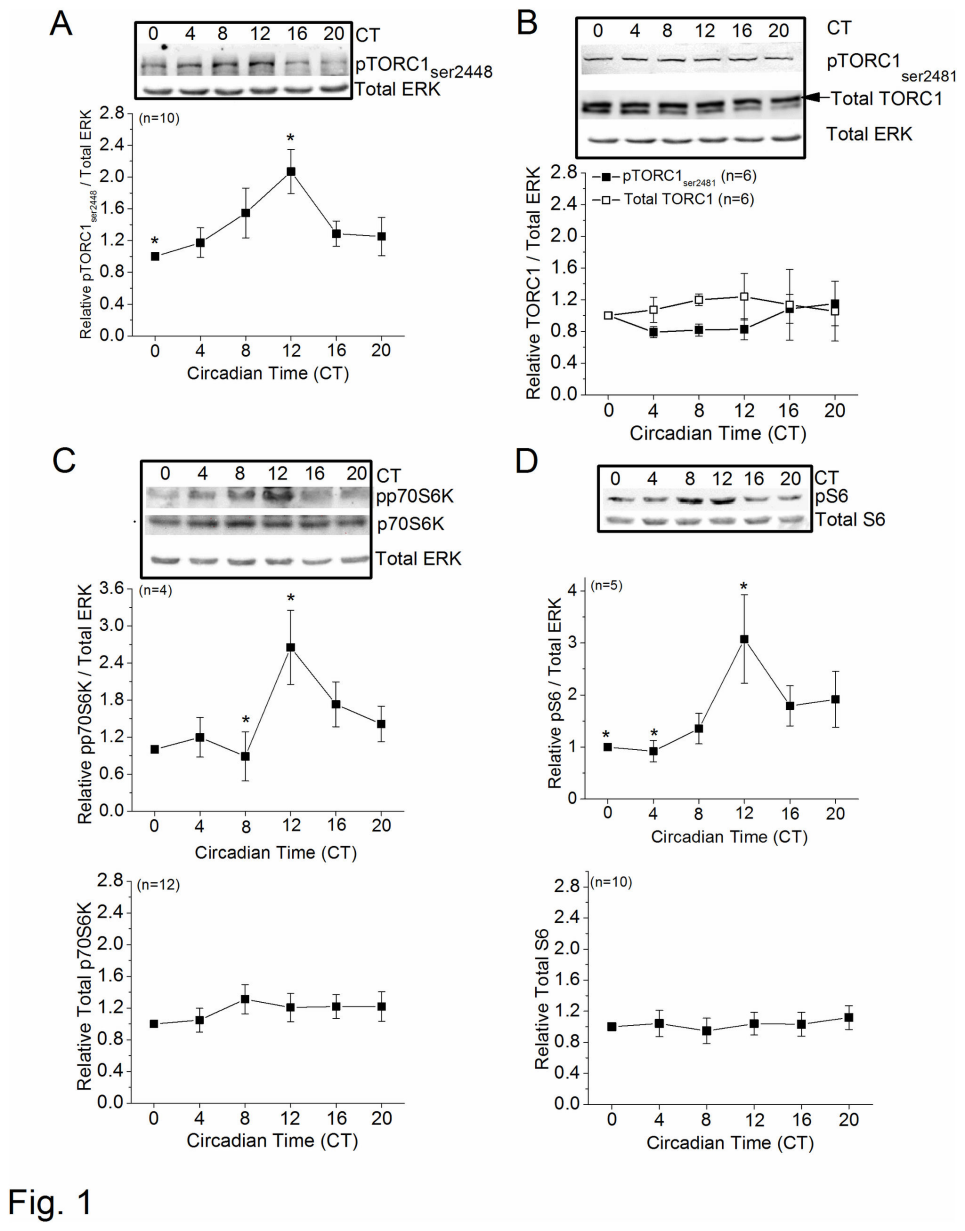
mTORC1 signaling is under circadian control. On the second day of DD after circadian entrainment to 12: 12 hr LD cycles for 7 days *in ovo*, intact retinas were collected at 6 different circadian time points (CT 0, 4, 8, 12, 16, and 20) for immunoblotting analysis. (A) Phosphorylation of mTORC1 at ser2448 (pTORC1_ser2448_) showed a circadian rhythm with its peak at CT 12. (B) The total amount of mTORC1 (total TORC1) and mTORC1 phosphorylation at ser2481 (pTORC1_ser2481_) did not display circadian rhythmicity. (C) Phosphorylation of the downstream target of mTORC1, p70-S6 kinase 1(pp70S6K), displayed a circadian rhythm with a peak at CT 12 (upper panel), while total p70S6K (lower panel) remained constant. (D) Phosphorylation of S6 ribosomal protein (pS6), the downstream target of p70S6 kinase, exhibited a circadian rhythm with a peak at CT 12 (upper panel), with total S6 protein (lower panel) constant throughout the day. * indicates a statistical significance at CT 12 compared to CT 0, CT4, or CT8. *p<0.05.

mTORC1 signaling participates in light-induced phase-shifts in mammals, as well as in the changes of circadian period in *Drosophila* [21–23]. We first investigated whether mTOR signaling was under circadian control in the avian retina, since the phosphorylation states of mTORC1 signaling display circadian rhythms in the suprachiasmatic nucleus (SCN) [47]. Chicken retinal samples were collected at six different circadian time (CT) points on the second day of DD after LD entrainment and used for Western blotting analysis. Since the total amount of ERK (total ERK) is constant throughout the day [37], we used total ERK as the loading control. We found that the phosphorylation status of mTORC1 was under circadian regulation, and it was a site-specific regulation. The phosphorylation at ser 2448 of mTORC1 (pTORC1_ser2448_), the site that monitors mTORC1 activity [48,49], peaked at CT 12 (Figure 1A), but phosphorylation at ser2481 (pTORC1_ser2481_) on the regulatory domain, as well as total mTORC1, remained constant (Figure 1B). Downstream of mTORC1, the phosphorylation states of p70S6 kinase (pp70S6K) and S6 ribosomal kinase (pS6) were also rhythmic with peaks at CT 12 (Figure 1C, 1D), while total p70S6K and S6 protein remained constant. Hence, the activation state of mTORC1 signaling was under circadian regulation in the retina.

### mTORC1 is involved in the circadian regulation of L-VGCC currents

**Figure 2:**
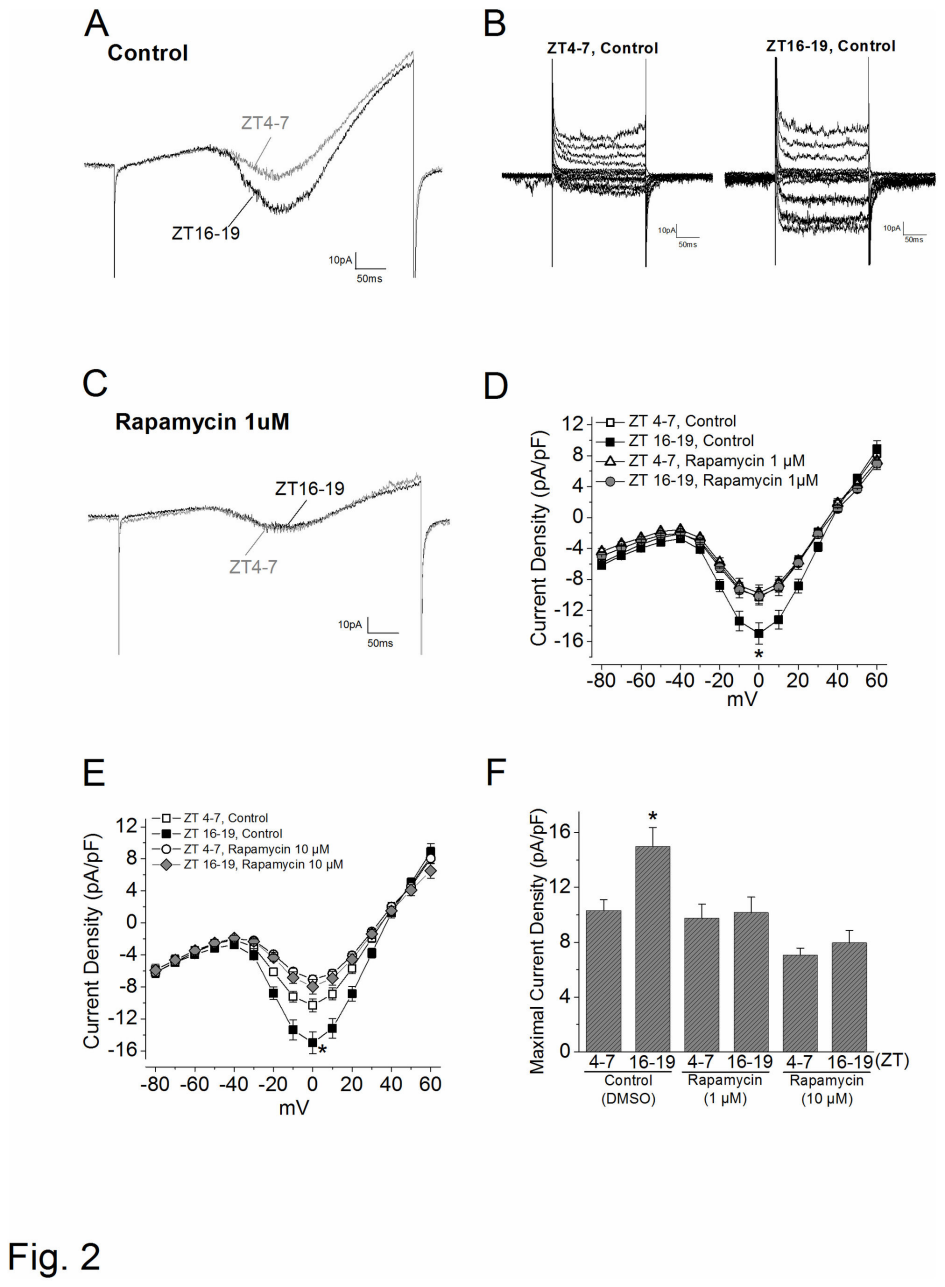
mTORC1 inhibitor dampens the circadian rhythm of L-VGCC currents. L-VGCCs were recorded from cultured chick cone photoreceptors on the sixth day of LD entrainment during the day (ZT 4-7) or at night (ZT 16-19). (A) Representative day (ZT 4-7) and night (ZT 16-19) control L-VGCC current traces from cells treated with 0.1% DMSO; (A) ramp command, (B) step commands. (C) Two representative traces from cells that were treated with rapamycin (1 µM) for 2 hr prior to recordings. (D) and (E) The average current-voltage (I–V) relationships are shown in current density (pA/pF) and step-voltage (mV). * indicates that the control group at ZT 16-19 is significantly different from the other groups. (F) The maximal current densities were elicited at 0 mV. * indicates that the L-VGCC current density recorded at night (control, ZT 16-19; n=23) is significantly higher than those recorded during the day (control, ZT 4-7; n=21), rapamycin treated cells recorded during the day (ZT 4-7; 1 µM n=16; 10 µM n=13) and night (ZT 16-19; 1 µM n=18; 10 µM n=13). * indicates that the L-VGCC current density recorded from the control cells at night (control, ZT16-19) is significantly higher than all other groups. *p<0.05.

**Figure 3:**
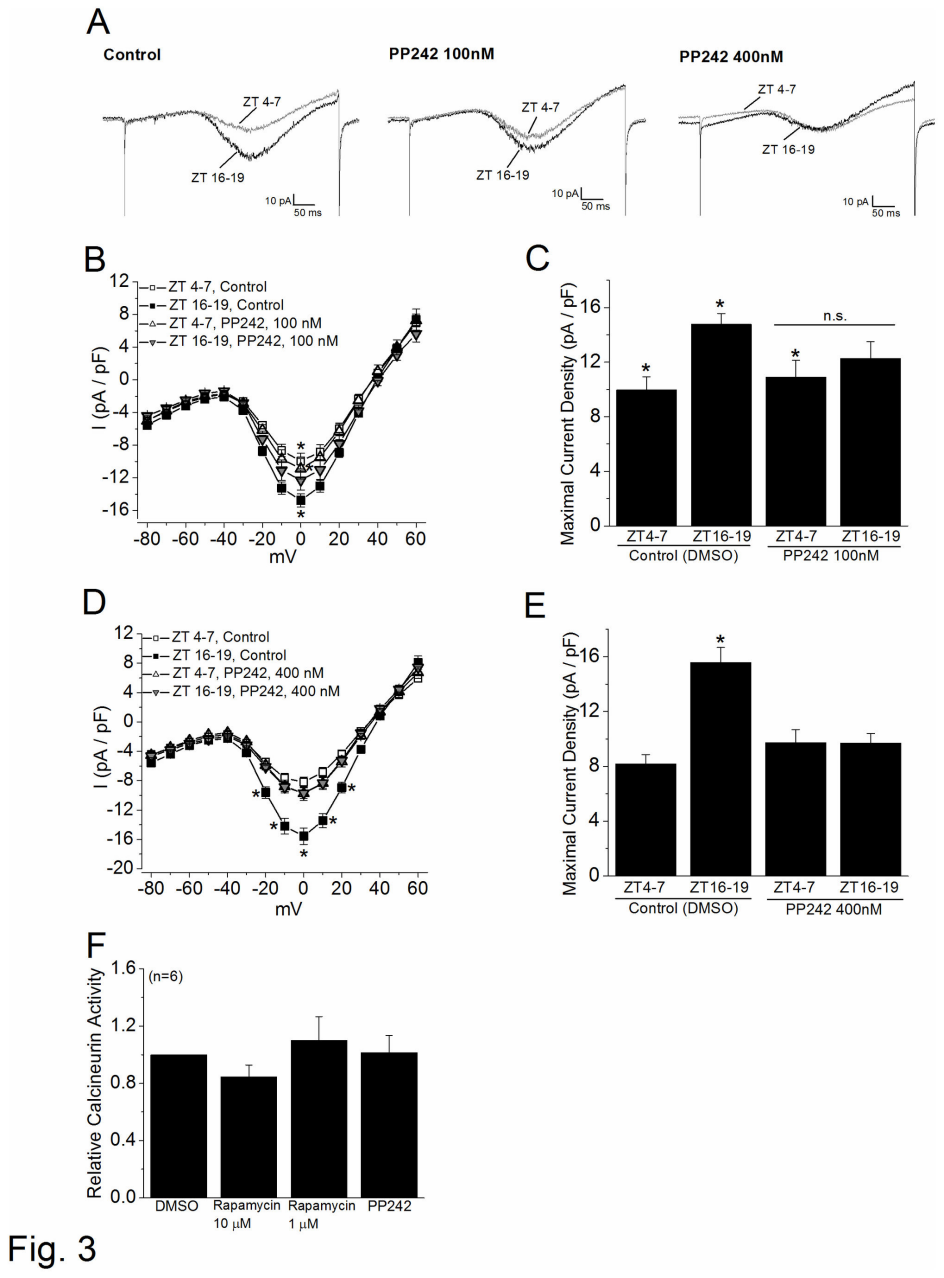
mTOR signaling regulates the circadian phase-dependent modulation of L-VGCCs. (A) Representative L-VGCC current traces from cells treated with 0.1% DMSO (control) or PP242 (100 nM or 400 nM) are shown. (B) The average current-voltage (I–V) relationships from cells treated with 0.1% DMSO (control) or PP242 (100 nM) are shown in current density (pA/pF) and step-voltage (mV). (C) The maximal current densities were elicited at 0 mV of the step command. * indicates that the L-VGCC current density of the controls recorded at night (ZT 16-19) is significantly higher than control and PP242 (100 nM) treated cells recorded during the day (ZT 4-7). PP242-treated cells that were recorded at night (ZT 16-19) have no statistical difference (n.s.) in L-VGCC current densities when compared to PP242-treated cells recorded during the day (ZT 4-7) or the controls recorded at night (ZT 16-19). Each group had at least 15 cells. (D) The average current-voltage (I–V) relationships from cells treated with 0.1% DMSO (control) or PP242 (400 nM) are shown in current density (pA/pF) and step-voltage (mV). * indicates that the L-VGCC current density of the controls recorded at night (ZT 16-19) is significantly higher than the other three groups. (E) Treatment with PP242 at 400 nM significantly dampened the circadian rhythm of maximal L-VGCC current densities. Each group had at least 15 cells. * indicates that the L-VGCC current density of the control group at night (ZT 16-19) is significantly higher than the other three groups. (F) Treatment with either rapamycin (10µM, 1µM) or PP242 (400nM) does not inhibit calcineurin activity compared to the control (0.1% DMSO) cultures. n=6 for each group. *p<0.05.

In the retina, the L-VGCCs are essential in neurotransmitter release from photoreceptors and other retinal neurons [39], and these channels are under circadian control in cone photoreceptors [38] and bipolar cells [50]. The maximal currents of L-VGCCs elicited at 0 mV are significantly larger at night than during the day (Figure 2A, 2B [38]). To investigate whether mTORC1 was involved in the circadian regulation of L-VGCCs, we applied rapamycin to inhibit mTORC1 in the following experiments. Rapamycin forms a complex with tacrolimus (FK506) binding protein 12 (FKBP12), an intracellular receptor protein, to inhibit mTORC1 activity (mTORC1 is more sensitive to rapamycin inhibition than mTORC2 [51]). After 2 hr treatment of rapamycin (1 µM), the L-VGCC current densities in cone photoreceptors were significantly dampened at night (ZT 16-20; Figure 2C, 2D), while there was no effect on currents recorded during the daytime (ZT 4-8; Figure 2C, 2D). Similar results were observed at a higher rapamycin concentration (10 µM; Figure 2E, 2F). To verify our observations were truly caused by the inhibition of mTOR, we applied PP242, another mTOR inhibitor not related to rapamycin structurally or mechanistically. PP242 inhibits both mTORC1 and mTORC2 by competing for ATP binding sites [52]. Treatment with PP242 (400 nM; 2 hr) significantly decreased the circadian rhythm of L-VGCC current densities at night (Figure 3A, 3D, 3E). Similar results were obtained at lower concentrations (100 nM) where PP242 inhibits mTORC1 more specifically than mTORC2 [52] (Figure 3A, 3B, 3C). Therefore, mTOR signaling caused a circadian phase-dependent modulation of L-VGCCs in chick cone photoreceptors, in which inhibition of mTOR significantly reduced L-VGCC currents at night.

We previously also demonstrated that calcineurin, a ser/thr phosphatase, regulates the L-VGCCs in a circadian phase-dependent manner, where inhibition of calcineurin dampens L-VGCC currents at night [45]. Rapamycin and FK506 are structurally related immunosuppressants that inhibit the lymphocyte-activation pathway through binding to FKBP12 [53–56]. While the rapamycin-FKBP12 complex inhibits mTORC1 signaling, the FK506-FKBP12 complex targets calcineurin [57]. Because of the structural similarities between rapamycin and FK-506 [58–60], we further verified that the action of rapamycin was not though interference of calcineurin activity at the concentrations used in this study. We examined calcineurin activity after cultured retinal cells were treated with rapamycin (10 µM or 1 µM), PP242 (400 nM), or DMSO (0.1%, control) for 2 hrs. At these concentrations, rapamycin and PP242 did not inhibit calcineurin activity (Figure 3F), consistent with other reports [61,62], while FK-506 clearly inhibits calcineurin activity as we and others have previously shown [45,61]. Hence, we used 1 µM rapamycin and 400 nM PP242 to inhibit mTORC signaling for the following experiments.

### mTORC1 is involved in the circadian regulation of L-VGCCα1D protein expression and channel trafficking

**Figure 4:**
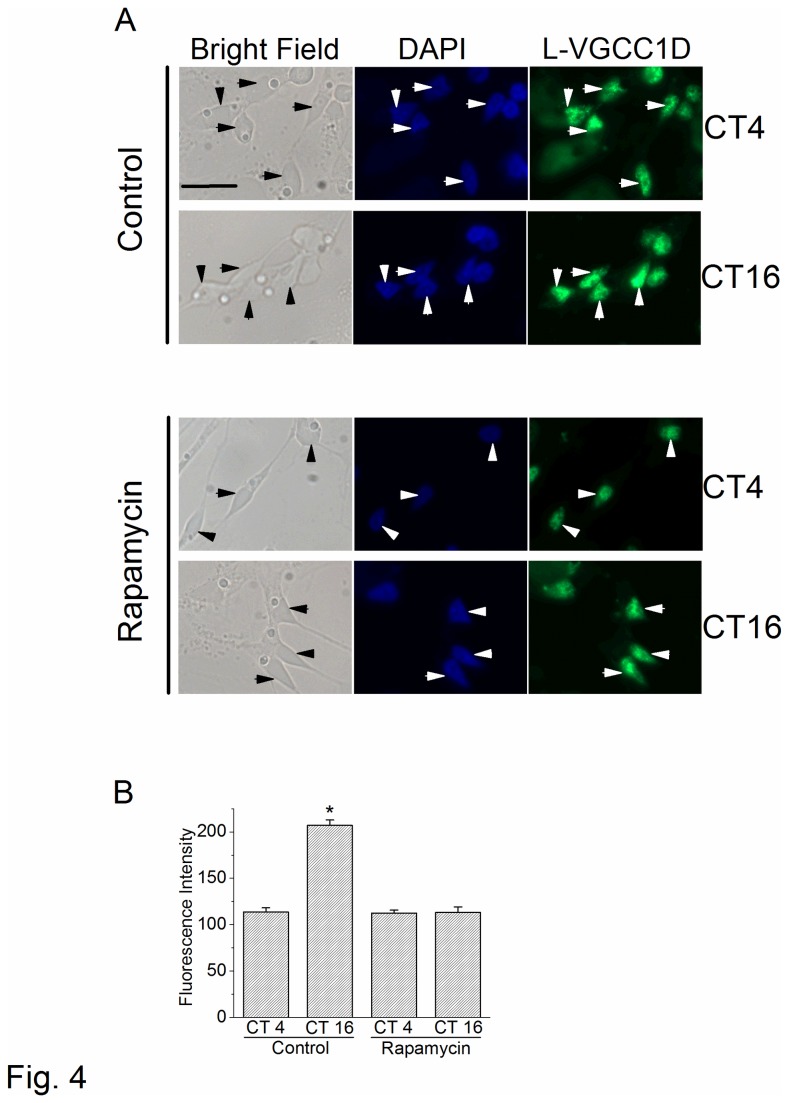
mTORC1 inhibition dampens the circadian rhythm of L-VGCCα1D. Retinal cells were cultured on glass coverslips and entrained to 12 hr LD cycles for four days in vitro and kept in DD. On the second day of DD, cells were treated with rapamycin at CT 2 and CT 14 for 2 hr followed by fixation at CT 4 and CT 16. After washing and blocking, cells were processed for L-VGCCα1D immunofluorescent staining. (A) Epifluorescent photos from the control (top panel) and rapamycin treated (lower panel) groups. The photographs shown here were slightly over-exposed to provide clearer images but were not used for statistical analysis in (B). The arrowheads indicate the cone photoreceptors. (B) The fluorescence intensity of L-VGCCα1D was significantly higher at CT 16 (control) compared to all other groups. Each group has at least 15 cells from 4 different trials. *p<0.05.

**Figure 5:**
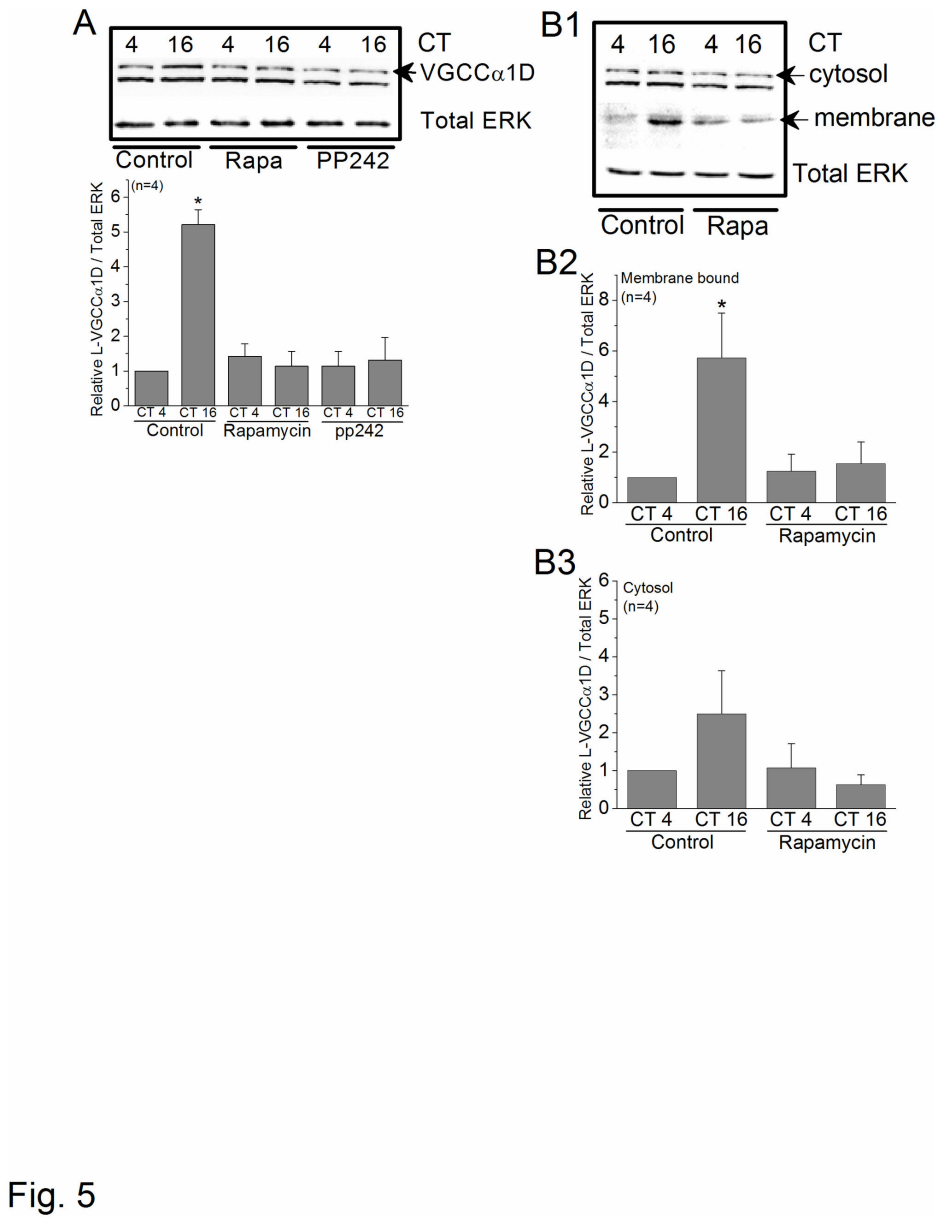
Inhibition of mTORC1 causes a circadian phase-dependent decrease in the protein expression and plasma membrane distribution of L-VGCCα1D. Chick embryos (E11) were entrained in LD cycles for 7 days *in ovo*, and retinas were dissected, cultured, and kept in DD. On the second day of DD, cultured retinal cells were treated with 0.1% DMSO (control), rapamycin, or PP242 for 2 hr prior to harvest at CT 4 and CT 16. (A) In the whole cell lysate, the total protein expression of L-VGCCα1D was significantly higher at night (CT 16) of the control compared to all other groups. Treatment with either rapamycin or PP242 dampened the circadian rhythm of L-VGCCα1D protein levels (B1-B3). After treatment with DMSO (control) or rapamycin for 2 hr, the distribution of L-VGCCα1D on the plasma membrane or cytosol was analyzed using biotinylation assays (B1). Representative blots of L-VGCCα1D from the cytosolic compartment (cytosol) and membrane-bound fraction (membrane). Total ERK served as loading control (B2). In the membrane-bound fraction, the L-VGCCα1D was significantly higher in control cells harvested at CT 16 compared to other groups (B3). In the cytosolic compartment (cytosol), there was no difference in the quantity of L-VGCCα1D among all groups. *p<0.05.

Since mTORC1 was involved in the circadian phase-dependent modulation of L-VGCC currents in cone photoreceptors, we next examined whether mTORC1 affected the protein expression of L-VGCCα1D and its trafficking from the cytosol to the plasma membrane. In the mammalian retina, the distribution of L-VGCCα1D in photoreceptors is wide-ranging from the inner segment layer, outer nucleus layer, and outer plexiform layer [63]. In avian cone photoreceptors, L-VGCCα1D is concentrated in the inner segment (including soma, Fig. 4 [38]). There was a significantly higher L-VGCCα1D fluorescence intensity in cone photoreceptors when cultures were fixed at CT 16 compared to CT 4 (Fig. 4A, top panel, Fig. 4B). Treatment with rapamycin for 2 hr significantly decreased the fluorescence intensity of L-VGCCα1D in photoreceptors fixed at CT 16 (Fig. 4A, lower panel, Fig. 4B). Using Western blot analysis, we found that retinal cultures treated with rapamycin or PP242 for 2 hr decreased the protein expression of L-VGCCα1D when cultures were harvested at night (CT 16; Figure 5A). We further used biotinylation assays to differentiate plasma membrane-bound versus cytosolic L-VGCCα1D and found that inhibition of mTORC1 significantly decreased the plasma membrane-bound L-VGCCα1D when cells were harvested at night (CT 16; Figure 5B1, 5B2). These results indicated that mTORC1 participated in the circadian regulation of protein expression and translocation of L-VGCCα1D. Since physiologically functional ion channels have to be transported and inserted into the plasma membrane first, these results echo the earlier data that mTORC1 is involved in the circadian regulation of L-VGCC currents (Figures 2, 3).

### mTORC1 is a downstream target of the PI3K-AKT pathway

**Figure 6:**
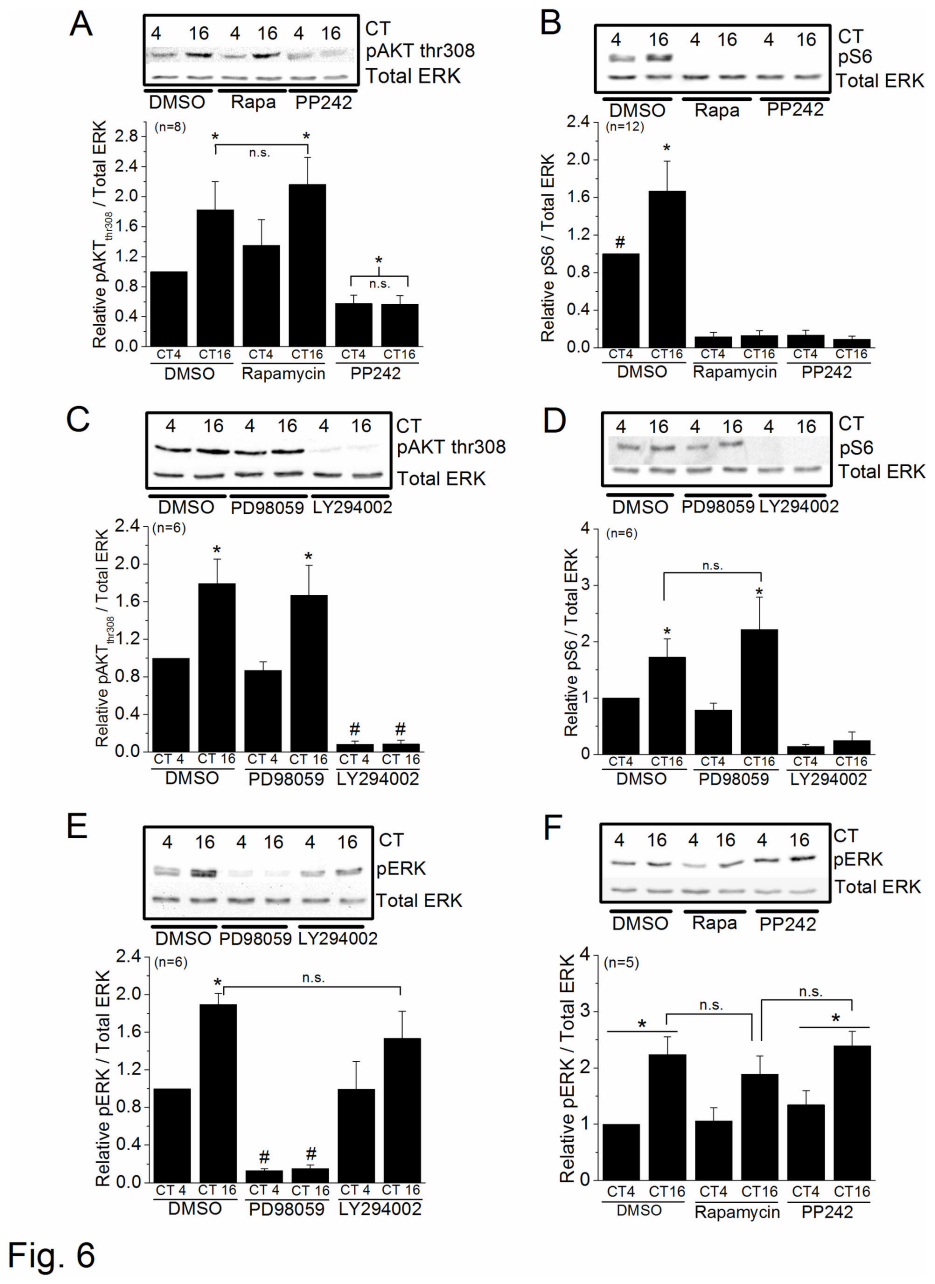
The mTORC1 pathway is downstream of PI3K-AKT. Chick embryos (E11) were entrained in LD cycles for 7 days *in ovo*, and retinas were dissected, cultured, and kept in DD. On the second day of DD, cultured retinas were treated with 0.1% DMSO (control) or various inhibitors for 2 hr prior to harvest for immunoblotting at CT 4 and CT 16. (A) The phosphorylation of AKT at thr308 (pAKT_thr308_) was significantly higher in the control and rapamycin treated cells at CT 16 compared to other groups. “n.s.” indicates that there is no statical difference between the two CT 16 groups. * indicates statistical differences. (B) The phosphorylation of S6 (pS6), a downstream target of mTORC1, is significantly higher in the control at CT 16 (*) compared to all other groups. The level of pS6 in the control at CT 4 (#) is significantly higher than groups treated with rapamycin or PP242. (C) The levels of pAKT_thr308_ are significantly higher in the control at CT 16 (*) and PD98059 treated group at CT 16 (*) compared to all other groups. The levels of pAKT_thr308_ are significantly lower in both groups (CT 4 and CT 16) treated with LY294002 (#). (D) The levels of pS6 are significantly higher in the control at CT 16 (*) and PD98059 treated group at CT 16 (*) compared to all other groups. (E) The level of di-phosphorylated ERK (pERK) is significantly higher in the control at CT 16 (*), and there is no statistical difference between the control at CT 16 and LY294002 treated group at CT 16. The pERK levels in both PD98059 treated groups (CT 4 and CT 16) are significantly lower (#) compared to all other groups. (F) Treatment with rapamycin or PP242 does not affect the circadian rhythm of pERK, in which the pERK level is significantly higher at night (CT 16, *) compared to the day time level (CT 4). *, ^#^ *p*<0.05.

We previously showed that the circadian regulation of L-VGCCs is in part through the regulation of channel trafficking [40]. The MAPK-ERK and PI3K-AKT signaling pathways are involved in ion channel translocation in neurons or cardiomyocytes [40,64,65], and both are downstream of Ras and parallel to each other in the circadian regulation of L-VGCC trafficking [40]. The phosphorylation/activation states of ERK and AKT are also under circadian control in the retina [37,38,40]. More specifically, the di-phosphorylation of ERK (pERK) and phosphorylation of AKT at thr308 (pAKT_thr308_) are significantly higher at night than during the day [38,40,45,66]. Since mTORC1 was involved in the circadian phase-dependent modulation of L-VGCCα1D trafficking and translocation (Figures 4 and 5), we next investigated whether there was any cross-talk between mTORC1 and MAPK-ERK or PI3K-AKT signaling. As parallel pathways, inhibitors that block MAPK-ERK signaling do not affect the circadian rhythm of pAKT and vice versa. We found that treatment with rapamycin did not affect the circadian rhythm of pAKT_thr308_ (Figure 6A) or pERK (Figure 6F). Treatment with PP242 inhibited pAKT_thr308_ both at night (CT 16) and during the day (CT 4; Figure 6A) but did not affect pERK (Figure 6F). The effect of PP242 on pAKT might be due to its non-specific inhibition of both mTORC1 and mTORC2, since AKT is a known downstream target of mTORC2 [14,67]. As a positive control, both rapamycin and PP242 completely abolished the phosphorylation of S6 (pS6), a direct downstream target of mTORC1 (Figure 6B). When cultured retinas were treated with PD98059 (50 µM), a MEK1 inhibitor, the circadian rhythm of pAKT_thr308_ (Figure 6C [40]) and pS6 (Figure 6D) were not affected, but the phosphorylation of ERK (pERK) was completely inhibited since MEK1 is directly upstream of ERK (Figure 6E). When cultured retinal cells were treated with the PI3K inhibitor LY294002 (50 µM), the phosphorylation of AKT _thr308_ (Figure 6C) and S6 (Figure 6D) were completely abolished but did not affect the circadian rhythm of pERK (Figure 6E [40]). Through these pharmacological studies, we concluded that mTORC1 was downstream of PI3K-AKT, but independent from MAPK-ERK signaling, to regulate the circadian rhythm of L-VGCCs.

## Discussion

**Figure 7:**
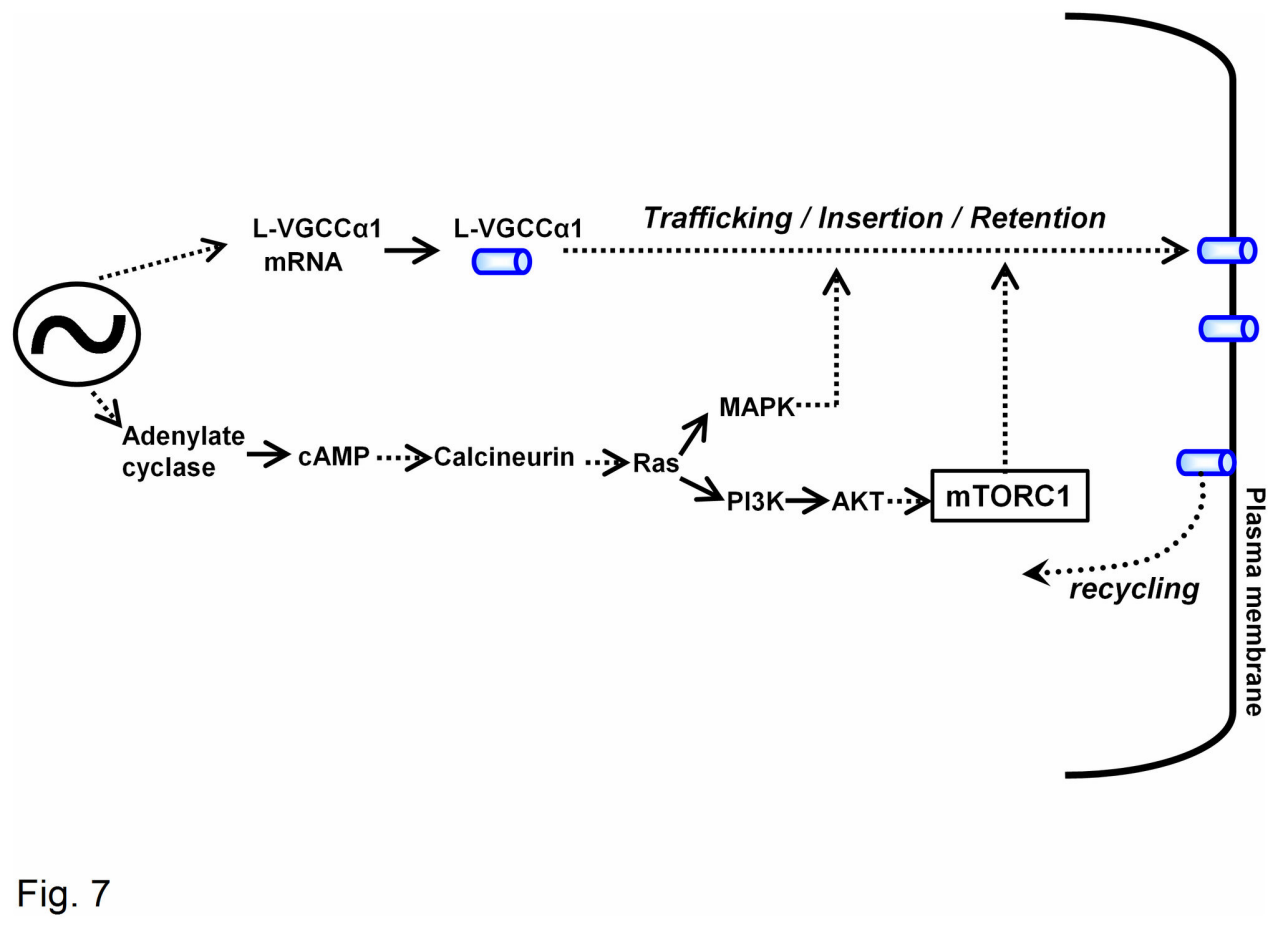
A schematic model summarizes the complex cell signaling in the circadian regulation of L-VGCCs. The circadian clock in the photoreceptor regulates the mRNA levels and protein expression of the channel forming L-VGCCα1 subunits. The circadian clock also regulates the activities / phosphorylation states of cell signaling molecules. In part, the complex cell signaling involves the trafficking, translocation, plasma membrane insertion, and/or membrane retention of L-VGCCα1. This model is based on our previous observations [37,38,40,45,66], as well as the current results.

Through four independent lines of investigation (electrophysiology, immunofluorescent staining, Western blotting, and surface biotinylation assays), we uncovered a new functional role for mTORC1, the circadian regulation of ion channels in cone photoreceptors. Inhibition of mTORC1 caused a circadian phase-dependent decrease of L-VGCC currents, as well as the distribution of L-VGCCα1D in the plasma membrane. Our results suggest that mTORC1 in part was involved in the channel trafficking and translocation from the cytosol to the plasma membrane, membrane insertion, and/or membrane retention of L-VGCCα1D. This conclusion was based on our previous observation, as well as others, that the PI3K-AKT pathway is involved in ion channel trafficking [40,64,65]. Since mTORC1 is downstream of PI3K-AKT (Figures 6 and 7), it is reasonable to conclude that in part, mTORC1 is involved in the circadian regulation of L-VGCC trafficking. Since we showed that L-VGCCs are more abundant in the plasma membrane at night compared to the day [40] (Figure 5B), mechanisms involved in the circadian regulation of L-VGCC plasma membrane insertion, membrane retention [68], or recycling are all possible actions of mTORC1, which will require further investigation.

The phosphorylation state of mTORC1-dependent signaling, but not its total protein expression, was also under circadian control. mTORC1 can be activated by phosphorylation at ser2448 [48,49], while ser2481 is an autophosphorylation site in the regulatory domain [69]. We found that the phosphorylation of mTORC1 at ser2448 (pTORC1_ser2448_), but not ser2481 (pTORC1_ser2481_), displayed circadian rhythm with a peak at CT 12, which indicated that the activity of mTORC1 was under circadian control, and the circadian regulation of mTORC1 was phosphorylation-site specific. The activation of p70S6 kinase (p70S6K) is pTORC1_ser2448_ dependent [10], which further phosphorylates S6 to initiate other cellular processes [70]. We showed that the phosphorylation of p70S6K (pp70S6K) and S6 (pS6) in the retina also displayed circadian oscillations in synch with pTORC1_ser2448_.

Since we previously characterized the circadian regulation of L-VGCCs is in part through both MAPK-ERK and PI3K-AKT signaling [38,40,45], we further deciphered whether there was any cross-talk among mTORC1, MAPK-ERK, and PI3K-AKT signaling. PI3K-AKT signaling activates mTORC1 phosphorylation, while mTORC2 is upstream of PI3K-AKT [71]. Even though MAPK-ERK signaling may also stimulate the mTORC1 dependent pathway [72], we did not observe any cross-talk between them in the retina. Through a series of pharmacological studies, we found that mTORC1-dependent signaling was downstream of PI3K-AKT and independent from MAPK-ERK (Figure 7). Hence, mTORC1-dependent signaling served as part of the circadian output pathway to regulate L-VGCCs in the retina.

mTORC signaling participates in many cellular processes including protein and lipid synthesis, metabolism, cell survival, growth [71], and is an important regulator of ribosome biogenesis [73]. When cellular energy levels are high, mTORC signaling promotes energy expenditure in processes such as protein translation and prevents autophagy [74]. When cells are under stress or nutrient-deprived conditions, mTORC signaling has the opposite action [75]. In the retina, activation of mTORC delays retinal cell death and promotes axon regeneration [16–20], but its inhibition results in the loss of cone photoreceptor opsins and retinal degeneration [17]. Therefore, mTORC promotes survival and neural protection. In addition, mTORC1 signaling is known to be involved in the circadian rhythms of both vertebrates and invertebrates [21–23]. The mammalian master circadian clocks are located in the suprachiasmatic nucleus (SCN) of the hypothalamus [76]. The phosphorylation state of mTORC1-dependent signaling displays circadian rhythms in the mouse SCN [47]. mTORC1 activity is light-inducible and involved in light-dependent circadian phase-shifting [22,23]. Disruption of mTORC1 signaling alters the light-induced expression of *Period* gene, a core component of the molecular clock, in the SCN [22]. In *Drosophila* , activation of mTORC1 impacts the nuclear accumulation of the circadian oscillator genes and lengthens the period of circadian oscillations [21]. Hence, mTORC1 plays an important role in regulating circadian rhythms.

In the present study, we discovered that mTORC1 signaling was involved in the circadian regulation of L-VGCCs in part through promoting L-VGCCα1D subunit translocation into the plasma membrane at night, and the activation of mTORC1 signaling was also under circadian control. Retinal photoreceptors are non-spiking neurons [39], and many of their intracellular processes including calcium homeostasis are highly compartmentalized [77]. In the dark, calcium influx through L-VGCCs at the synaptic terminals allows for the continuous release of neurotransmitters from the ribbon synapses [78]. In response to various light intensities, the phototransduction cascade and changes in local intracellular calcium take place in the outer segment [79–81]. Hence, calcium plays different roles in different localized compartments of photoreceptors.

In mammalian and avian photoreceptors, L-VGCCα1D is mainly distributed in the inner segment, soma, and synaptic terminals [38,63,82,83], where calcium is involved in the regulation of metabolism and neurotransmitter release [84]. While the circadian oscillators in photoreceptors regulate daily changes in various cellular processes, from gene and protein expressions [32–35,38] to light sensitivities [85], all of these processes are energy dependent. In addition, there are circadian regulations of both cGMP-gated cation channels [37,66,86] and L-VGCCs [38,40,45], which might ultimately regulate calcium homeostasis in photoreceptors. In vertebrates, glucose metabolism is under circadian control [87], and hence, the circulating plasma glucose that reaches the retina for neuronal fuel might be oscillating daily. We postulate that the circadian regulation of L-VGCCs through mTORC1 signaling might be essential to photoreceptor metabolism and energy expenditure, since metabolism and gene expression occur in the inner segment and the soma [84,88], where L-VGCCα1D is also heavily distributed. The circadian oscillation in mTORC1 activation and the circadian phase-dependent increase of L-VGCCs in the plasma membrane of inner segments and the soma would allow for local increases of calcium influx, which would further enhance calcium-dependent gene / protein expressions, potentially for subsequent needs in intersegmental transport [89], outer segment renewal [31], and energy requiring retinomotor movement [29,30,90]. Disruption of mTORC1 activation and L-VGCC circadian rhythm could further alter intracellular calcium homeostasis, which might lead to photoreceptor pathophysiological conditions and degeneration. In summary, we showed that the activation of mTORC1-dependent signaling was under circadian control, and the circadian rhythm of L-VGCCs in cone photoreceptors was in part through the PI3K-AKT-mTORC1 pathway. More specifically, mTORC1 participated in the circadian phase-dependent modulation of L-VGCCα1D trafficking and translocation. Hence, mTORC1 signaling is indispensable in maintaining healthy physiological function in the retina.
